# Topics of Public Interest

**Published:** 1912-02

**Authors:** 


### TOPICS OF PUBLIC INTEREST

Dr. Rupert Blue of the United States Public Health and Marine Hospital Service, having been promoted by the President, assumed charge as Surgeon-General, January 12, 1912. While, in a sense, the failure of the scheme to place an outsider in charge of the service may be attributed to numerous protests of professional bodies, we believe that the President's conception of honor and of the fitness of things, would have sufficed. It is now the duty of the profession to get after the medical politicians who concocted this scheme and to deal with them in such a thoroughly disagreeable way that there will be no danger of a repetition of the experiment. Candidates for the three government medical services are subjected to an examination which weeds out from 75 to 90 per cent. of those applying, and which deters the vast majority from applying at all. The appointees are held to a rigid discipline, are promoted only on examination as to intellectual, executive and physical fitness, are paid only moderately, in proportion to the demands made upon them. One of the implicit compensations of the service, is the chance of rising to the generalship. To take this chance from tried men who have grown up in the service, would be dishonorable.

### Examination of Dentists for the U. S. Army.

EXAMINATIONS for the appointment of Acting Dental Surgeons will be held at Fort Slocum, New York; Columbus Barracks, Ohio; Jefferson Barracks, Missouri; Fort Logan, Colorado; and Fort McDowell, California, on Monday, April 1, 1912. Blanks and information can be procured by addressing the "Surgeon General, U. S. Army, Washington, D. C." Blanks must be returned at least two weeks before the examination.

After three years' service at $150.00 per month if found qualified, appointees are promoted to the grade of dental surgeon with the rank of first lieutenant.

---

THE Lockport Academy of Medicine has adopted an advanced schedule of rates: $2.00 for day calls, $3.00 for night calls, $15.00 for what the newspapers very modestly term family cases. Much popular dissatisfaction is expressed at the increase. This is natural enough. The ultimate unit of value is the day's labor. Raise of wages is of no avail unless prices remain as before. If a man has accumulated the equivalent of a million day's wages and the wage, expressed in dollars is doubled, half of the capitalists' accumulation has been confiscated. When the accumulated wealth is large enough, such confiscation means no perceptible decrease in comfort of life. Hence the tendency is to confiscate from the small business and professional man, in the interests of the laboring man.

---

UNITED STATES CIVIL SERVICE EXAMINATIONS.—A great variety of positions, clerical, mechanical and professional, in widely different services, at home and in our possessions, are to be filled. The following appeal most directly to our readers: Physician and Matron, Indian Service, Veterinarian Philippine Service, March 13. Pharmacist, P. H. & M. H. Service, Scientific Assistant, Department of Agriculture, Trained Nurse, Indian Service, April 10.

We would advise anyone who can do anything reasonably well and who desires government employment, to inquire of the United States Civil Service Commission, Washington, promptly.

---

THE Young Women's Christian Association of Buffalo is undertaking a campaign to raise $50,000 for the improvement of the main building, the lease of quarters for transients near the depot, and the purchase of a summer home on the lake shore. These are worthy reasons, offered by a philanthropy of tried and proved worth. The second object seems to us especially important, in view of the gross injustice, sometimes ruin, done to strange girls by ostensibly kind-hearted acquaintances.

---

The American Academy of Medicine, a national organization of college educated physicians, devoted to educational and sociologic matters, announces a conference on Conservation of School Children, at S. Bethlehem, Pa., Wednesday and Thursday, April 3 and 4, 1912. The profession is invited to attend and, so far as eligible, to become members. Lehigh University will afford meeting places and extend its hospitality in various ways. Children are almost as deserving of conservation as streams and trees and we trust that the profession will cooperate heartily.

---

The New York State Civil Service Commission (address at Albany for particulars and application blanks), will hold an examination for candidates for junior physicianships in the State Hospitals for the Insane, February 24. The position is open to both Regulars and Homoeopaths, and is not limited to residents of the state. The salary is $900 and maintenance, increased annually by $100. till the limit of $1200 is reached, after which there is a chance for promotion.

---

Mrs E. H. Harriman will defray the expense of an investigation as to Public Health, Hospitals and Budget, under a committee of the New York Academy of Medicine.

---

Tonawanda's typhoid incidence for 1911 was almost double that of 1910. Dr. W. A. Scott, Surgeon of Co. E, 3d Reg. N. G. N. Y., will inoculate as many of the men as desire it.

